# Synthesis of 7,12-bis(4-(di(1H-pyrrol-2-yl)methyl)phenyl)benzo[k]fluoranthene from a new dialdehyde as a novel fluorometric bis-Dipyrromethane derivative

**DOI:** 10.3906/kim-2004-72

**Published:** 2021-02-17

**Authors:** Faride RANJBARI, Salar HEMMATI, Mohammad Reza RASHIDI

**Affiliations:** 1 Faculty of Pharmacy, Tabriz University of Medical Sciences, Tabriz Iran; 2 Drug Applied Research Centre, Tabriz University of Medical Sciences, Tabriz Iran

**Keywords:** Dipyrromethanes, benzo[k]fluoranthene, bis-dipyrromethane, aldehyde, pyrrole, fluorescent

## Abstract

Dipyrromethanes are useful mediator structures which can be used as a part of other molecules such as bis-porphyrins and their derivations. Various methods have been developed for their synthesis. This study presents the synthesis of a new bis-dipyrromethane, 7,12-bis(4-(di(1H-pyrrol-2-yl)methyl)phenyl)benzo[k]fluoranthene, using the Lewis acid catalyzed reaction between a new dialdehyde and pyrrole at room temperature. The UV spectroscopic and fluorometric properties of the final product and precursors were determined. The newly synthesized product with desirable UV spectroscopic and fluorometric properties has the potential to be applied as a part of bis­porphyrins or it can be used for other purposes in future studies.

## 1. Introduction

Dipyrromethanes (DPMs) are among the major intermediates used for the synthesis of porphyrins or porphyrin analogues [1]. Given the difficulties in transsubstituted porphyrins synthesis and purification, the synthesis of DPMs has been studied using the group focusing on the designed porphyrin [2]. Hence, different analogues of porphyrins and related compounds (dipyrrins, calixpyrroles, chlorins, and corroles) could be prepared by the condensation of aldehydes and DPMs [3–4]. Porphyrins are particularly important for their photophysical and photochemical properties, and their synthesis is under development [5–7].

More recently, other applications of DPMs have received growing attention, with new studies exploring the methodology or the new product synthesis of these compounds. In fact, DPMs are precursors to the preparation of BODIPY dyes, which are applied as high-performance imaging probes due to their fluorescent properties [8]. Another application of these compounds is concerned with their use in the development of new optical anion sensors. Also, they can be utilized as photonic organic-based materials [7–11].

Over the past decade, there have been advances in the synthesis methods of DPMs [1,12]. Some of these developments are as follows: (i) synthesis by condensation of excess pyrrole and aldehyde or ketone with Lewis or Brønsted acid as the catalyst (e.g., trifluoroacetic acid (TFA) [11,13], BF_3_·Et_2_O [14] or InCl3[15]), (ii) synthesis of the unsymmetrical DPM from α-acylpyrroles [16], and (iii) synthesis by the reaction of ending alkynes and pyrrole with In(OTf)3 as the catalyst [17]. The idea of bis-DPMs synthesis from diformyl compounds was originally presented by Sessler [18], McLendon [19], Osuka [20], Lindsey [2], and other researchers during the 1980s. Bis-DPMs are mostly synthesized with the condensation of a bis-DPM and two DPMs and aldehydes to be used in bis-porphyrins structures. Recently, Hongbin Zhao et al. have reported some new conjugated bis-DPMs. These compounds contain various arylene linkers which could be a part of multiple porphyrins [21]. Other designs and applications of bis-DPM were presented by Sessler et al. in 2018 [22]. They synthesized a formylated DPM, pyrene-functionalized tetrakis-(1H-pyrrole-2-carbaldehyde), and investigated its properties as an anion receptor. Polycyclic aromatic hydrocarbons (PAHs) refer to a large class of organic chemistry with different sizes and structures [17]. In light of these differences, they have found extensive applications including liquid crystals in optoelectronics or conducting materials in solar cells [23–26]. The benzo[k]fluoranthenes represent another group of interesting PAH compounds. Special properties of these compounds are due to the modification of peripheral functional groups which make them a suitable candidate for a variety of purposes, including luminescent materials, molecular sensors, or organic semiconductors [27–30].

In the present study, a new highly conjugated bis-DPM, 7,12-bis(4-(di(1H-pyrrol-2-yl)methyl)phenyl)benzo[k]fluoranthene was designed and synthesized with new benzo[k]fluoranthenes which served as a linkage for a new corresponding dialdehyde. The fluorescence properties of the resultant compound were then evaluated. Due to its mild synthesis procedure and efficient UV spectroscopic and fluorometric properties, this compound can be considered as a promising candidate for bis- porphyrin synthesis or other DPMs purposes.

## 2. Material and methods 

### 2.1. Materials

All reactions were conducted under inert atmosphere, and the solvents were dried using the standard methods. Substrate for the synthesis, 1,2- acenaphthoquinone, 4-bromophenylaceticacid, pyrrole, trifluoroacetic acid (TFA), acetic acid, p-methyl benzaldehyde, TsOH, 2,3-Dichloro-5,6-dicyano-1,4-benzoquinone (DDQ), pyridine,1,3-Dicyclohexylcarbodiimide (DCC), anthranilic acid, BF_3_.Et_2_O, butyllithium, and POCl_3_ were purchased from Merck and Aldrich. 

### 2.2. Methods

The ^1^H and ^13^CNMR spectra were measured with a Bruker FT-400 and 300 MHz (^13^CNMR: 100 and 75 MHz) spectrometers at room temperature using CDCl_3_ as the solvent. The UV-Vis spectroscopy was performed using Shimadzu UV-2550. Fluorometry analysis was conducted using the Jasco Spectrofluorometer FP 8300. The ESI mass spectra were obtained using Agilent Technologies 5975C and Waters Alliance Series LC. The FT-IR spectra were recorded with FT-IR-8400S Shimadzu. Elemental analysis was carried out using Costech instruments and the elemental combustion system (See supporting information).

### 2.3. Synthesis

#### 2.3.1. Synthesis of 1,3-bis(4-bromophenyl)propan-2-one (1)

Synthesis of **1** was conducted as described in the literature [31]. Compound 1 was obtained as a white solid with a yield of 54% and Rf = 80% polar. m.p. 115–118 °C, Anal. Calcd. for C_15_H_12_Br_2_O(368.93): C, 48.95; H, 3.29; Br, 43.42; O,4.35. Found: C, 48.75; H, 3.41.

#### 2.3.2. Synthesis of 7,9-bis(4-bromophenyl)-8H-cyclopenta[a]acenaphthylen-8-one (2)

Synthesis of **2** was performed according to the published description in the literature [32]. The mixture containing 2.09 g (5 mmol) of compound 1 and 0.91 g (5 mmol) of acenaphthylene-1,2-dione was heated up to the reflux temperature in dry ethanol. Then, the reaction was sustained by adding a solution of KOH (1.5 M) over 5 min. The reaction color changed to dark green after 10 min and it refluxed for 24 h. For the purpose of purification, the flask content was filtered and the crude product was washed with cold toluene several times to yield 30% of compound 2 as a dark green solid. m.p. 400–405 °C; Anal. Calcd. for C_27_H_14_Br_2_O(514.22): C, 63.07; H, 2.74; Br, 31.08; O,3.11. Found: C, 63.21; H, 2.88.

#### 2.3.3. Synthesis of 7,12-bis(4-bromophenyl)benzo[k]fluoranthene [33] (3)

The solution of compound **2** , 0.88 g (1.7 mmol), was heated under reflux in dry 1,2-dichloroethane (35 mL) inside a three-necked flask equipped with two adding funnels. Then, anthranilic acid 0.779 g (5.5 mmol) dissolved in 10 mL of 1,2-dichloroethane (taken from one- necked flask) and Iso-amyl nitrite 0.644 g (5.5 mmol) diluted in 10 mL of 1,2-dichloroethane (taken from the another necked flask) (both solutions from each adding funnel) were added dropwise simultaneously over 45 min. The reaction was continued for 1 week. The mixture solvent was evaporated and purified with column chromatography (silica, toluene / n-Hexane, 8:2) to yield 98% of compound 3 as a yellow solid. Rf = 95% polar, first spot on the TLC, m.p. 254–256 °C; ^1^HNMR (300MHz, CDCl_3_, ppm): δ 6.72–6.74 (d, J=6.9 Hz, 2H, Ph), 7.37–7.47 (m, 8H, Ph), 7.59–7.60 (2H, m, Ph), 7.74–7.77 (m, 2H, Ph), 7.82–7.84 (m, 4H, Ph), ^13^CNMR (75 MHz, CDCl_3_, ppm): δ 121.13, 121.25, 122.24, 123.25, 123.36, 125.28, 125.46, 126.90, 127.08, 127.50, 128.99, 130.73, 130.82, 131.44, 131.51, 132.58, 132.88, 132.97, 133.68, 134.77, 136.08, 136.17, 137.59, 137.69, 137.79; ESI Mass: m/z 563 (calcd. for [M + H]^+^ 563; Anal. Calcd. for C_32_H_18_Br_2_ (562.30): C, 68.31; H, 3.23; Br, 28.42. Found: C, 68.59; H, 3.12.

#### 2.3.4. Synthesis of 4,4’-(benzo[k]fluoranthene-7,12-diyl)dibenzaldehyde [34-35](4)

One gram (1.7 mmol) of **3** solved in dried THF in a 100-mL three-necked round-bottom flask equipped with stirrer and septum under inert atmosphere was reacted with 3 mL (4 mmol, 1.4M) of butyllithium, which was added dropwise for a period of 10 min at –78 °C (the temperature was maintained with ethyl-acetate and liquid nitrogen). After the solution changed color to yellow, 6 mL (excess) DMF was infused into the flask (from the septum). The mixture was stirred for 1 h and then hydrolyzed by adding reaction content to 200 mL solution (2M) of HCl. The hydrolysis process lasted 4 days. The yellow compound was purified with column chromatography (silica) with elution of a gradient of solvents (starting from n-Hexane to n-Hexane- ethyl-acetate (9:1)) to yield 45% of compound 4 as a yellow solid. (R_f_= 30% polar, third spot on the TLC). m.p. 304 – 307 °C; ^1^HNMR (300MHz, CDCl_3_, ppm): δ 6.61–6.63 (d, J = 6.3 Hz, 2H, Ph), 7.32–7.37 (t, J1= 7.5, J2= 8.1 Hz, 2H, Ph), 7.4–7,5 (m, 2H, Ph), 7. 5–7.6 (m, 2H, Ph), 7.74–7.80 (t, J1= 8.4, J2= 8.1 Hz, 4H, Ph), 7.77 (d, J= 6.6,4H, Ph), 10.28 (s, 2H, COH), ^13^CNMR (75 MHz, CDCl_3_, ppm): δ 121.25, 123.23, 125.20, 125.44, 126.91, 127.46, 129.59, 129.67, 129.88, 129.96, 131.71, 132.02, 132.10, 134.54, 135.83, 136.37, 145.49, 190.91, 193.18; ESI Mass: m/z 461 (calcd. for [M + H]^+^ 461); Anal. Calcd for C_34_H_20_O_2_ (460.53): C, 88.67; H, 4.38; N, 6.95. Found: C, 88.84; H, 4.26; N, 7.18.

#### 2.3.5. Synthesis of 7,12-bis(4-(di(1H-pyrrol-2-yl)methyl)phenyl)benzo[k]fluoranthene [36] (5)

The reaction of **4** , 0.2 g (4 mmol) and 4.7 g of pyrrole (70 mmol) was carried out in dried dichloromethane under inert atmosphere of nitrogen in the presence of 0.02 g (0.17 mmol) BF_3_.Et_2_O as a catalyst for 30 min. The catalyst was quenched by adding 2 mL solution of NaOH (0.1 M), and was then washed with water. The crude compound of dark orange was purified by thin-layer chromatography (silica) and n-hexan–ethylacetate (8:2) as eluent to yield 45% of compound 5 as an orange solid. (R_f_ = 60 % polar, the first spot on the TLC) , m.p > 360 °C decomposed; ^1^HNMR (400MHz, CDCl_3_, ppm): δ 5.72 (2H, s, CH (aliphatic)),6.06–6.12 (m, 4H, Pyrrole (CH β)), 6.26–6.28 (dd, J1= 2.76, J2= 2.82 Hz, 2H, Pyrrole (CH β)), 6.82–6.83 (dd, J1= 2.52, J2= 2.55 Hz, 4H, Pyrrole (CH α)), 7.32–7.35 (t, J1= 3.28, J2= 3.32 Hz, 2H, Ph), 7.39–7.41 (m, 2H, Ph), 7.52–7.53 (m, 8H, Ph), 7.64–7.66 (dd, J1= 7.8, J2= 7.4 Hz, 2H, Ph), 7.70–7.72 (m, 2H, Ph), 8.16 (s.broad of NH (Pyrrole)); ^13^CNMR (100MHz, CDCl_3_, ppm): δ 43.00, 106.44, 107.60, 116.50, 121.15, 122.96, 123.45, 124.78, 125.77, 126.77, 127.79, 128.25, 129.26, 129.87, 131.53; ESI Mass: m/z 693 (calcd. for [M + H]^+^ 693); Anal. Calcd. for C_50_H_36_N_4_ (692.87): C, 86.68; H, 5.24; N, 8.09. Found: C, 86.83; H, 5.43; N, 8.30.

##### 2.4. Spectroscopic property

###### 2.4.1. UV spectroscopic property

UV spectra were measured using the UV-Vis device (Shimadzu UV-2550) from freshly prepared compound solutions with a concentration of 0.5 mM in DMF at 300–700 nm bandwidth at room temperature.

###### 2.4.2. Fluorescence property

Excitation and emission spectra of three compounds (3,4, and 5) doped in DMF are shown in Figure. The fluorescence spectra were recorded at the fixed excitation wavelength of 310 nm and the emission range of 300–700 nm. For measuring the quantum yield of fluorescence the reported method for various compounds was used [37]. Amounts of quantum yield of fluorescence for new compounds were estimated related to the quinine sulfate as a standard. The compounds and the standard material were used with concentrations which showed less than 1% absorbance at 310 nm. Furthermore, the compounds with these concentrations were used for the evaluating of fluorescence properties. The following relationship can be used for estimating the quantum yield of fluorescence:

ΦX=ΦST(GradXGradST)(ηX2ηST2)

**Figure 1:**
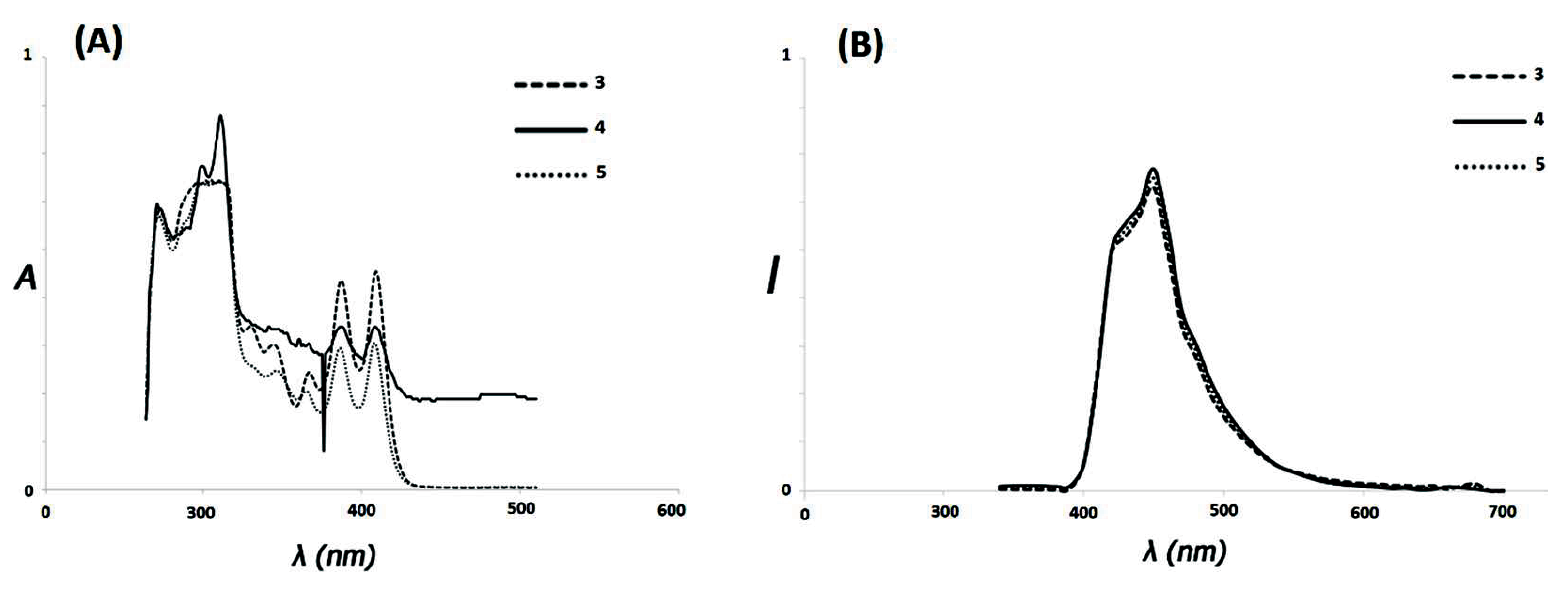
Normalized absorption (a) and fluorescence (b) emission spectra performed at ƛ_ex_= ƛ_max_(abs) of 3, 4, and 5 in DMF at room temperature.

The subscripts ST and X define standard and test, respectively, Φ is the fluorescence quantum yield, Grad the gradient from the plot of integrated fluorescence intensity vs absorbance, and η the refractive index of the solvent. 

## 3. Results

**Scheme 1:**
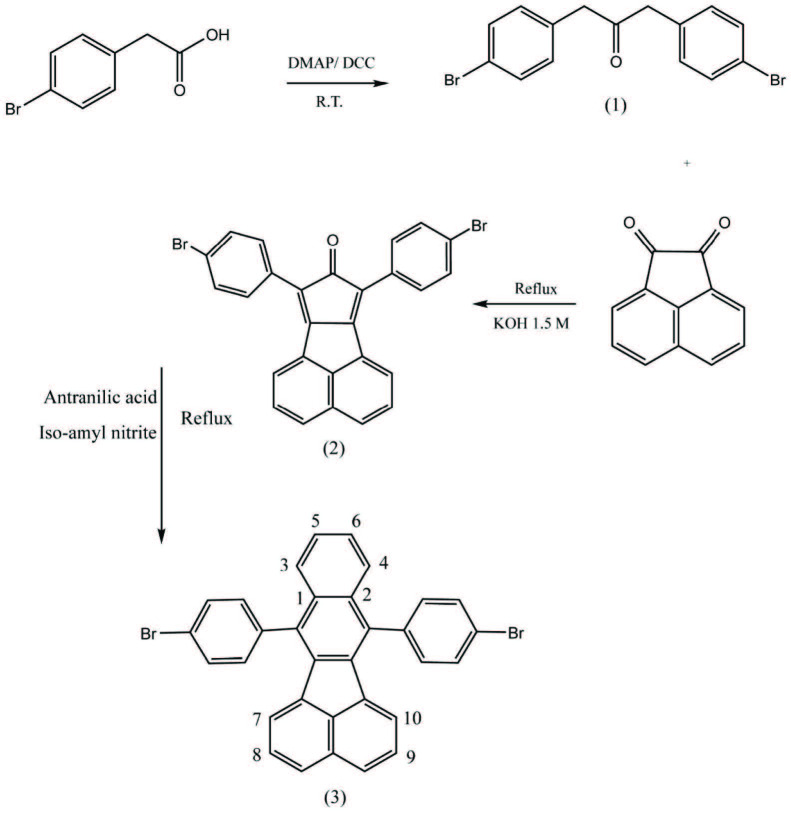
Synthesis of 7,12-bis(4-bromophenyl)benzo[k]fluoranthene.

**Scheme 2:**
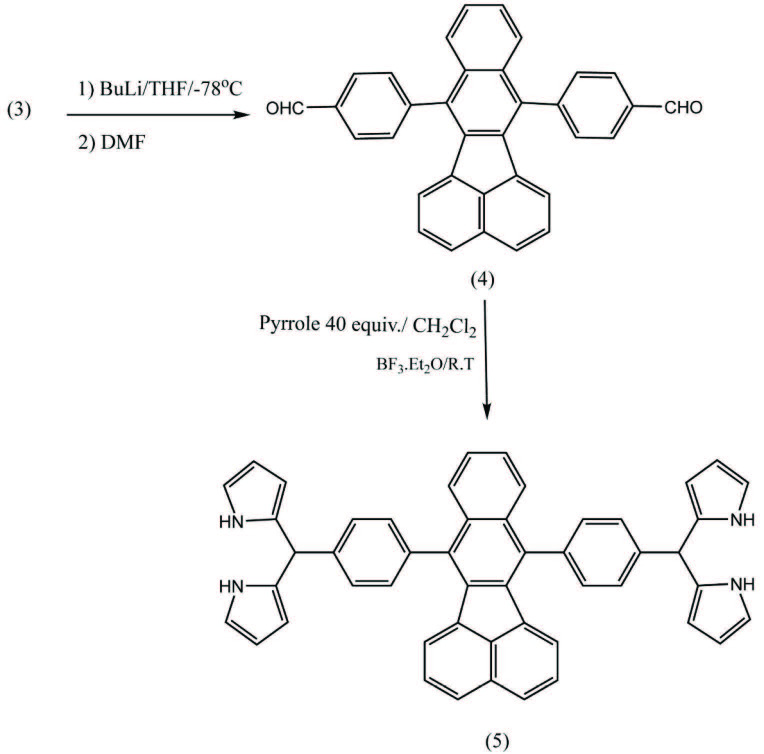
Synthesis of 7,12-bis(4-bromophenyl)benzo[k]fluoranthene (5).

7,9-bis(4-bromophenyl)-8H-cyclopenta[a]acenaphthylen-8-one (2) was synthesized in alkaline ethanol after 24 h of reflux [32]. A pure green compound was achieved after washing crude compound with cold toluene several times. The IR spectrum of this product indicated a strong peak at 1700 cm–1, which could be attributed to the carbonyl group. 7,12-bis(4-bromophenyl)benzo[k]fluoranthene (3) was prepared as a result of the reaction between 2 and amyl nitrite and anthranilic acid using the Diels-alder mechanism (via benzyne intermediate) [33]. At ^1^HNMR spectrum, two protons of H5 and H6 appeared at 6.72 and 6.74 ppm respectively. H3 and H4 could also be observed at 7.82 and 7.84 ppm respectively. Based on MM2 minimizing energy calculations conducted by Chemoffice 2016, the molecule structure of 3 is nonplanar and the end phenyls formed at angles with each other [38]. Therefore, H3 and H4 along with protons H7 and H10 are located in the magnetic field of ending phenyls. Due to anisotropic effects, protons located in the middle of a magnetic field will be shielding to fewer chemical shifts. Therefore, in ^1^HNMR spectrum of this structure, the peaks at 7.82–7.84 ppm belong to the above four protons and they are not real doublets. ^13^CNMR spectra showed signals of Benzo[k]fluoranthene section carbons of C6, C5, C4, C3, and C1,2 at 130.73, 130.82, 131.44, 131.51, and 132.88, respectively (Scheme 1). Moreover, the mass spectroscopy of the structure indicated the molecular weight of molecule 3 in 562. The 3D structure of molecule 3 is provided in the supplementary file. 4,4’-(benzo[k]fluoranthene-7,12-diyl)dibenzaldehyde (4) was synthesized by organometallic procedures [34,35] in extra dry THF at –78 °C. Purification was conducted using column chromatography. The IR spectrum showed an aldehyde carbonyl peak at 1710 cm–1 and C-H of aldehyde at 3200 cm–1 which confirmed successful formation of the new product (4). The ^1^HNMR spectrum indicated aldehyde hydrogen at 10.28 ppm. Moreover, the carbon of aldehyde carbonyl group was observed at 190.919 and 193.183 ppm in the ^13^CNMR. Regarding the mass spectroscopy, M+ appeared at 460 (Scheme 2).

7,12-bis(4-(di(1H-pyrrol-2-yl)methyl)phenyl)benzo[k]fluoranthene (5) was synthesized according to the procedure described earlier [39]. Compound 4 reacted with 40 equiv. pyrrole and a catalytic amount of BF_3_.Et_2_O at room temperature and in darkness for 30 min with CH2Cl2 (DCM) as the solvent. The TLC spot of the new product was formed a little below starting material 4. We once performed this reaction with TFA with Lewis acid and 70 equiv. pyrrole as the reactant and solvent but the reaction maintained with different by-products and low yields. To resolve this problem, we used milder Lewis acid. Since the new product was sensitive to being kept in solvents such as DCM, n-Hexane, ethyl acetate, and toluene for a long time, it needed to be purified as soon as possible. After purification of this compound, it should be used immediately in the next reaction or kept in the vacuum and darkness. N-H stretching at 3400 cm–1 in the IR spectroscopy showed substitution of DPM instead of aldehyde group. ^1^HNMR spectrum confirmed the formation of a new product (5) by displaying a broad peak of N-H at 8.1 ppm and the characteristic proton peak of dipyrromethane moiety at 5.7 ppm [39]. Also, the removal of the aldehyde hydrogen indicated that reaction proceeded from both sites. On the other hand, ^13^CNMR spectrum of this compound, which shows methine carbon at 43.006 ppm and α position carbon in pyrrole moiety at 116.5 ppm, confirmed conversion to 5.

The UV-Vis spectra of 4, 5, and 6 are shown in Figure. The maximum absorption of three compounds was measured at room temperature in DMF. Excitation and emission spectra of three compounds (3,4, and 5) evaluated at the fixed excitation wavelength of 310 nm in DMF are shown in Figure. The fluorescence spectra were recorded at the fixed excitation wavelength of 310 nm and the emission range of 300–700 nm. The maximum emission was observed at 450 nm in all three compounds with fluorescence intensities of 89791, 92560, and 95340 a.u., respectively. Therefore, the conjugation system improved fluorescence intensity from 4 to 6, but this change was not significant. The fluorescence quantum yield for the three compounds was determined in the chloroform relative to quinine sulfate dissolved in H2SO4 (0.1 M). Three compounds did not show a clear difference between fluorescence quantum yields; therefore, the long resonance chain had negligible effect on the fluorescence of these compounds [37,40]. Fluorometry data analysis is shown in Table.

**Table:** ^a^Absorption maximum in DMF. ^b^ Emission maxima in DMF. ^c^ Fluorescence quantum yield in chloroform determined relative to quinine sulfate in 0.1 H_2_SO_4_ as a standard (ƛ_ex_ = 310 nm).

compd	ƛ_max_ (nm) (logƐ)^a^	ƛem (nm)^b^	ɸ_f_^c^
3	273 (3.46), 310 (3.5), 393 (3.19), 413 (3.25)	450	0.57
4	273 (3.45), 310 (3.5), 393 (3.00), 413 (3.03)	450	0.57
5	273 (3.46), 310 (3.58), 393 (3.17), 413 (3.18)	450	0.58

## 4. Discussion

The new compound 5, Bis-dipyrromethane, was synthesized with a new dialdehyde and then examined for the UV spectroscopic and fluorescence properties.

Dipyrromethanes are significant intermediates due to their particularity in porphyrin and dye complexes with boron, which are used in imaging, anionic sensors, and other applications. In the majority of uses associated with dipyrromethanes, the UV spectroscopic properties and the fluorescence molecule absorption are important. Thus, we have focused out attention on a compound with high conjugation and valid fluorescence properties. 

In the UV spectroscopic studies conducted by Saranya et al. [40], benzo[k] fluoranthene and some derivatives have been examined. The molecules with structures and optical properties comparable to our compounds had great potentials to be used in organic light-emitting diodes (OLEDs). In another paper published by Han et al. [41], several Acenaphtho[1,2k]fluoranthen derivate were studied for their UV spectroscopic and fluorescence properties. They investigated some compounds with molecule properties similar to those used in our study. Furthermore, the estimated quantum yield of fluorescence potency in these compounds is lower than ours. However, the products had good potency in the nondoped OLED devices. In this paper, we have introduced a novel Bis-dipyrromathane with good spectroscopic and fluorescence properties which can be used in most of future researches. On the other hand, it should be noted that compound 5 is an intermediate, which can participate in a porphyrin structure and achieve an extensive conjugation by removing the methine proton. This extensive conjugation will have a positive effect on maximum absorption and fluorescence potency of the compound. High potentials for imaging could be achieved in the same manner where the compound 5 as an intermediate is converted into boron complex.

## 5. Conclusion

In sum, we introduced a novel Bis DPM and characterized with NMR and Mass spectroscopy. Also, we determined spectroscopic properties of new products. 

The novel bis-dipyrromethane, 5, with desirable UV spectrometric properties and fluorescence quantum yield could be used in other DPMs applications in the future studies. For example, compound 5 after being used in a bis-porphyrin structure [40], and oxidation and removal of methine proton in the porphyrin ring, would have more favorable spectroscopic properties. That is due to the long resonance chain between the two linked porphyrins caused by a highly conjugated linkage. 

Supplementary Materials Click here for additional data file. Full experimental detail, FT-IR Spectra, 1H and 13C NMR spectra and mass spectra, can be found in the “Supplementary Materials” section of this article.
