# Supplementary material for: Synthesis of 7,12-bis(4-(di(1H-pyrrol-2-yl)methyl)phenyl)benzo[k]fluoranthene from a new dialdehyde as a novel fluorometric bis-Dipyrromethane derivative
Source: Turk J Chem. 2021 Feb 17;45(1):42–9. doi: 10.3906/kim-2004-72 (PMC7925304; doi:10.3906/kim-2004-72)
Supplement: Supplementary file 1 — Supplementary Materials [file turkjchem-45-42-sup001.pdf]

SUPPLEMENTARY MATERIALS

MATERIALS AND METHODS

Solvents and reagents **Solvents and reagents**

All reactions were conducted under inert atmosphere, and the solvents were dried using the standard methods. Substrate for the synthesis, 1,2- acenaphthoquinone, 4-bromophenylaceticacid, pyrrole, Trifluoroacetic acid (TFA), acetic acid, p-methyl benzaldehyde, TsOH, 2,3-Dichloro-5,6-dicyano-1,4-benzoquinone (DDQ), pyridine,1,3-Dicyclohexylcarbodiimide (DCC), anthranilic acid, $BF_3.Et_2O$, Butyllithium, $POCl_3$ were purchased from Merck and Aldrich.

Spectra

The ^1H, $^{13}CNMR$ spectra were measured with a Bruker FT-400 and 300 MHz ($^{13}CNMR$: 100 and 75 MHz) spectrometers at room temperature and $CDCl_3$ as the solvent. The UV-Vis spectroscopy was performed by Shimadzu UV-2550. Fluorimetry analysis was conducted by Jasco Spectrofluorometer FP 8300. The ESI Mass spectra were obtained using Waters 2695 Separation Module and Waters Alliance Series LC. The FT-IR spectra were recorded with FT-IR-8400S Shimadzu. Elemental analysis was undertaken using Costech instruments and the elemental combustion system.

Procedures

Synthesis of bis(4-methylbenzyl)ketone (1)

Synthesis of **1** was conducted as described in the literature [1]. Compound **1** was obtained as a white solid with a yield of 54% and R_f= 80% polar. m.p. 115-118 °C, Anal. Calcd for $C_{15}H_{12}Br_2O$ (368.93): C, 48.95; H, 3.29; Br, 43.42; O,4.35. Found: C, 48.75; H, 3.41.

Synthesis of 7,9-bis(4-bromophenyl)-8H-cyclopenta[a]acenaphthylen-8-one (2)

Synthesis of **2** was performed according to the published as described in the literature [2]. The mixture containing 2.09g (5mmol) of compound **1** and 0.91 (5 mmol) of acenaphthylene-1,2-dione was heated up to the reflux temperature in dry ethanol. Then, the reaction was sustained by adding a solution of KOH (1.5 M) during 5 min. The reaction color changed to dark green after 10 min and it refluxed for 24 hours. For the purpose of purification, the flask content was filtered and the crud product was washed with cold toluene several times to yield 30% of compound **2** as a dark green solid. m.p.400-405°C; Anal. Calcd for $C_{27}H_{14}Br_2O$ (514.22): C, 63.07; H, 2.74; Br, 31.08; O,3.11. Found: C, 63.21; H, 2.88.

Synthesis of 7,12-bis(4-bromophenyl)benzo[k]fluoranthene [3] **(3)**

The solution of compound **2**, 0.88g (1.7mmol), was heated under reflux in dry 1,2-dichloroethane inside a three-necked flask equipped with two adding funnels. Then, anthranilic acid dissolved in 1,2-dichloroethane (taken from one- necked flask) and Iso-amyl nitrite diluted in 1,2-dichloroethane (taken from the another necked flask) (both solutions from each adding funnel) were added dropwise simultaneously during 45 min. The reaction was continued for one week. The mixture solvent was evaporated and purified with column chromatography (silica, toluene / n-Hexane, 8:2) to yield 98% of compound **3** as a yellow solid. R_f= 95% polar, first spot on the TLC, m.p. 254- 256 °C; ^{1}HNMR (300MHz, $CDCl_3$, ppm): δ 6.72- 6.74 (d, J=6.9 Hz, 2H, Ph), 7.37- 7.47 (m, 8H, Ph), 7.59- 7.60 (2H, m, Ph), 7.74- 7.77 (m, 2H, Ph), 7.82- 7.84 (m, 4H, Ph), ^{13}CNMR (75 MHz, $CDCl_3$, ppm): δ 121.13, 121.25, 122.24, 123.25, 123.36, 125.28, 125.46, 126.90, 127.08, 127.50, 128.99, 130.73, 130.82, 131.44, 131.51, 132.58, 132.88, 132.97, 133.68, 134.77, 136.08, 136.17, 137.59, 137.69, 137.79; ESI Mass**:** m/z 563 (calcd. for $[M + H]^+$ 563; Anal. Calcd for $C_{32}H_{18}Br_2$ (562.30): C, 68.31; H, 3.23; Br, 28.42. Found: C, 68.59; H, 3.12.

Synthesis of 4,4'-(benzo[k]fluoranthene-7,12-diyl)dibenzaldehyde [4-5] **(4)**

1g (1.7 mmol) of **3** solved in dried THF in a 100ml three necked round-bottom flask equipped with stirrer and septum under inert atmosphere reacted with 3ml (4mmol, 1.4M) of butyl lithium which added dropwise during 10min at -78°C (the temperature was maintained with ethyl-acetate and liquid nitrogen). After changing solution color to yellow, 6ml (excess) DMF was infused into the flask (from the septum). The mixture was stirred for 1h and then hydrolyzed by adding reaction content to 200ml solution (2M) of HCl. The hydrolysis process lasted 4 days. The yellow compound was purified with column chromatography (silica) with elution of a gradient of solvents (starting from n-Hexane to n-Hexane- ethyl-acetate (9:1)) to yield 45% of compound 4 as a yellow solid. (R_f= 30% polar, third spot on the TLC). m.p. 304- 307°C; ^{1}HNMR (300MHz, $CDCl_3$, ppm): δ 6.61- 6.63 (d, J=6.3 Hz, 2H, Ph), 7.32- 7.37 (t, J_1= 7.5, J_2= 8.1 Hz, 2H, Ph), 7.4- 7,5 (m, 2H, Ph), 7. 5- 7.6 (m, 2H, Ph), 7.74- 7.80 (t, J_1= 8.4, J_2= 8.1 Hz, 4H, Ph), 7.77 (d, J= 6.6,4H, Ph), 10.28 (s, 2H, COH), ^{13}CNMR (75 MHz, $CDCl_3$, ppm): δ 121.25, 123.23, 125.20, 125.44, 126.91, 127.46, 129.59, 129.67, 129.88, 129.96, 131.71, 132.02, 132.10, 134.54, 135.83, 136.37, 145.49, 190.91, 193.18; ESI Mass**:** m/z 461 (calcd. for $[M + H]^+$ 461); Anal. Calcd for $C_{34}H_{20}O_2$ (460.53): C, 88.67; H, 4.38; N, 6.95. Found: C, 88.84; H, 4.26; N, 7.18.

Synthesis of 7,12-bis(4-(di(1H-pyrrol-2-yl)methyl)phenyl)benzo[k]fluoranthene [6] **(5)**

The reaction of **4**, 0.2g (4 mmol) and 4.7g of pyrrole (70mmol) was carried out in dried dichloromethane under inert atmosphere of nitrogen in the presence of 0.02 g (0.17mmol) $BF_3.Et_2O$ as a catalyst for 30 min. The catalyst was quenched by adding 2ml solution of NaOH (0.1M), and then was washed with water. The crude compound of dark orange was purified by thin-layer chromatography (silica) and n-hexan–ethylacetate (8:2) as eluent to yield 45% of compound **5** as an orange solid. (Rf= 60 % polar, the first spot on the TLC) , m.p> 360°C decomposed; ^{1}HNMR (400MHz, $CDCl_3$, ppm): δ 5.72 (2H, s, CH (aliphatic)), 6.06- 6.12 (m, 4H, Pyrrole (CH β)), 6.26-6.28 (dd, J_1= 2.76, J_2= 2.82 Hz, 2H, Pyrrole (CH β)), 6.82-6.83 (dd, J_1= 2.52, J_2= 2.55 Hz, 4H, Pyrrole (CH α)), 7.32-7.35 (t, J_1= 3.28, J_2= 3.32 Hz, 2H, Ph), 7.39-7.41 (m, 2H, Ph), 7.52-7.53 (m, 8H, Ph), 7.64-7.66 (dd, J_1= 7.8, J_2= 7.4 Hz, 2H, Ph), 7.70-7.72 (m, 2H, Ph), 8.16 (s.broad of NH (Pyrrole)); ^{13}CNMR (100MHz, $CDCl_3$, ppm): δ 43.00, 106.44, 107.60, 116.50, 121.15, 122.96, 123.45, 124.78, 125.77, 126.77, 127.79, 128.25, 129.26, 129.87, 131.53;

ESI Mass: m/z 693(calcd. for $[M + H]^+$ 693); Anal. Calcd for $C_{50}H_{36}N_4$ (692.87): C, 86.68; H, 5.24; N, 8.09. Found: C, 86.83; H, 5.43; N, 8.30.

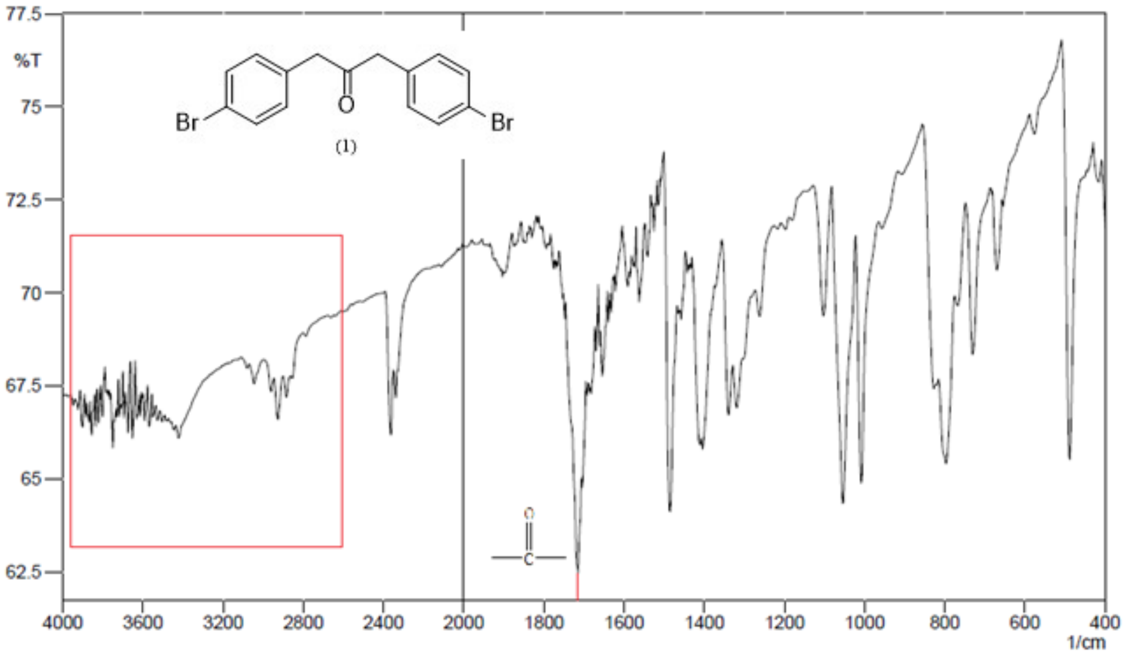

IR spectrum of bis(4-methylbenzyl)ketone (1)

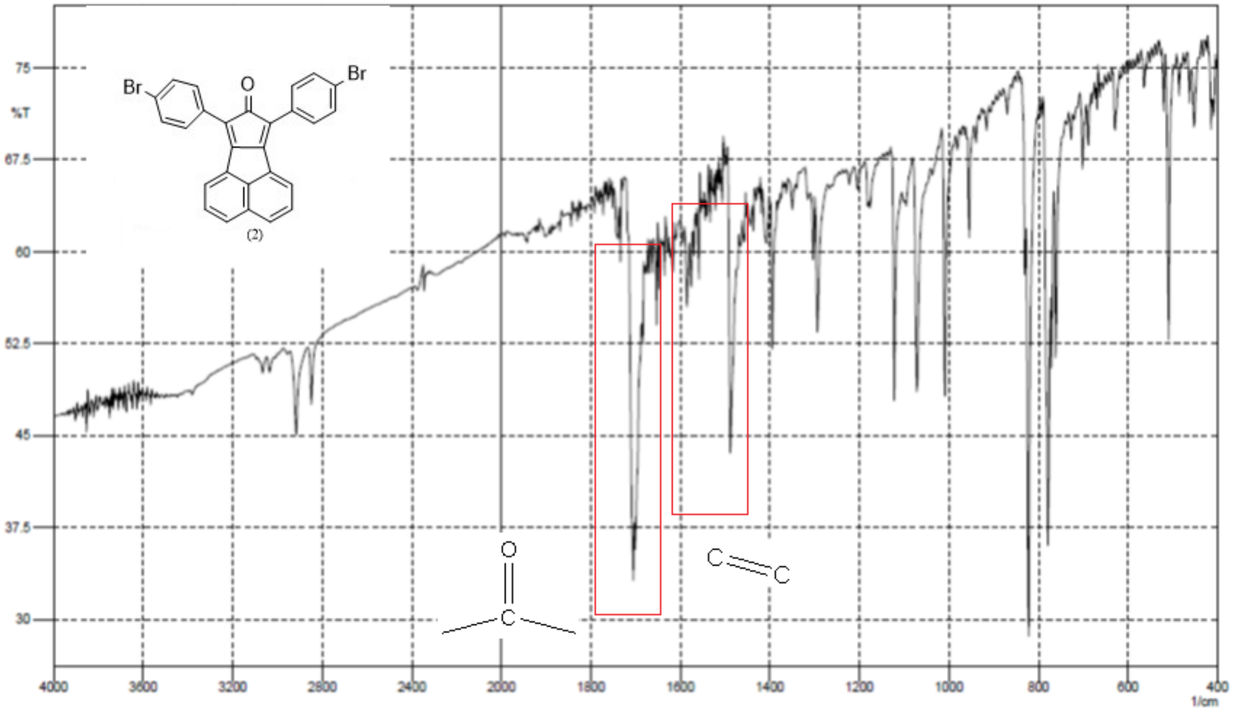

IR spectrum of 7,9-bis(4-bromophenyl)-8H-cyclopenta[a]acenaphthylen-8-one (2)

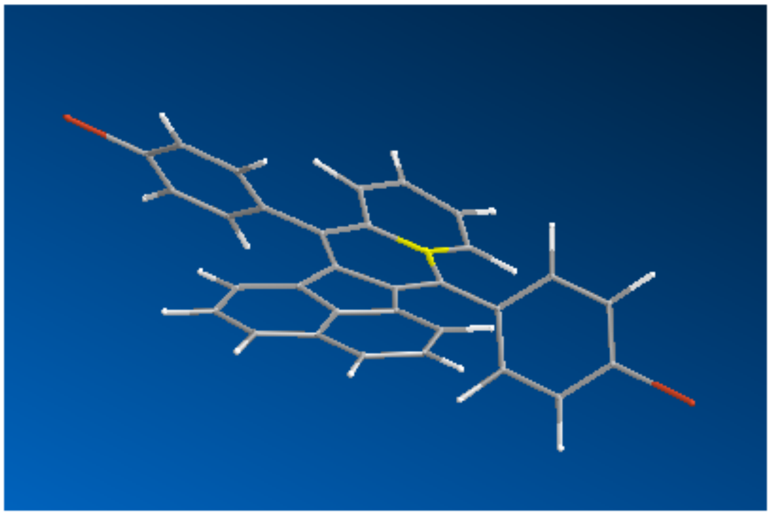

3D structure of **3**

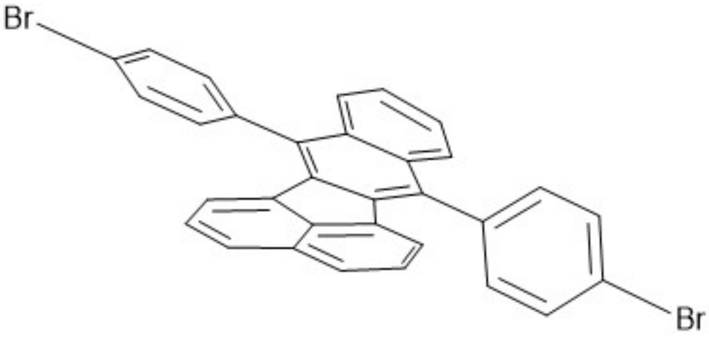

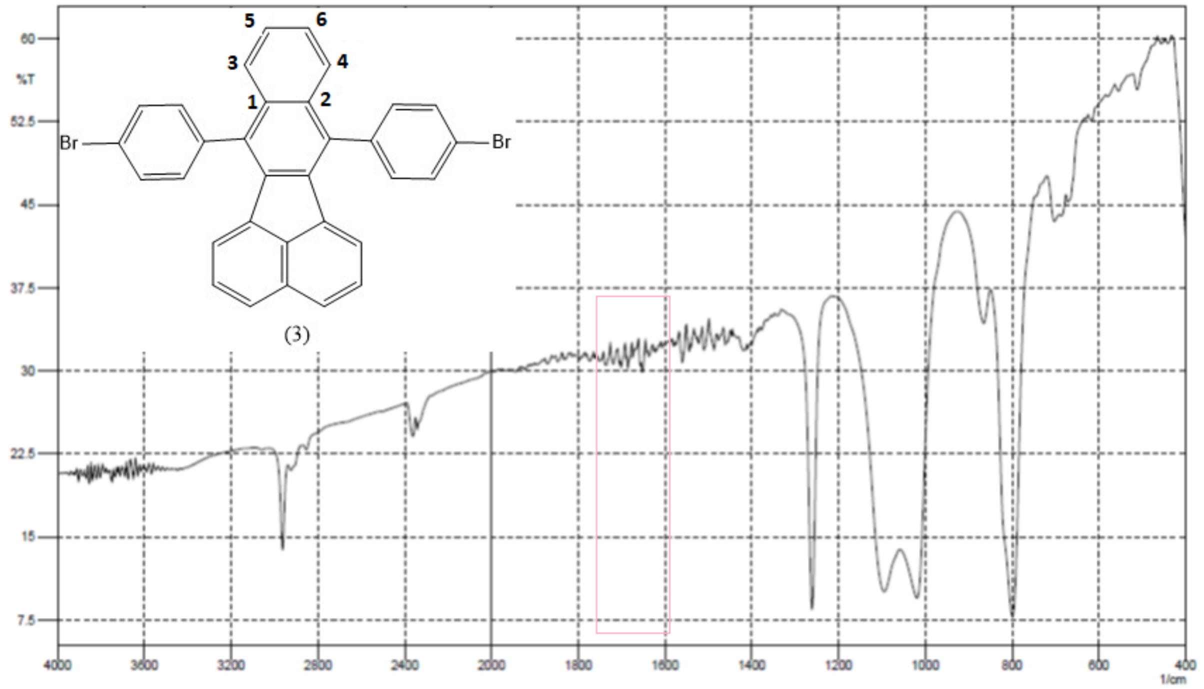

IR spectrum of 7,12-bis(4-bromophenyl)benzo[k]fluoranthene (**3**)

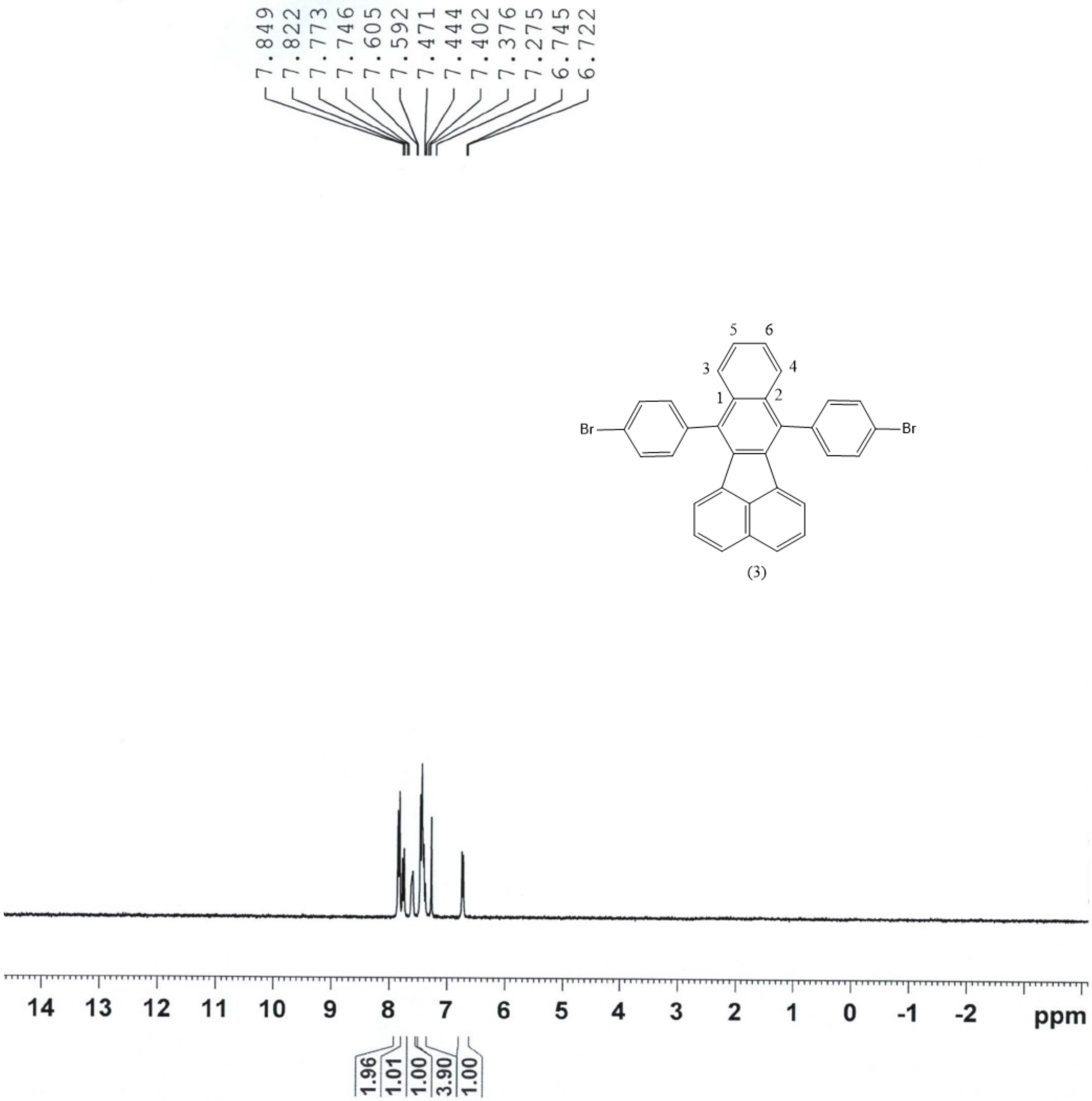

^{1}HNMR spectrum of **3** in $CDCl_3$

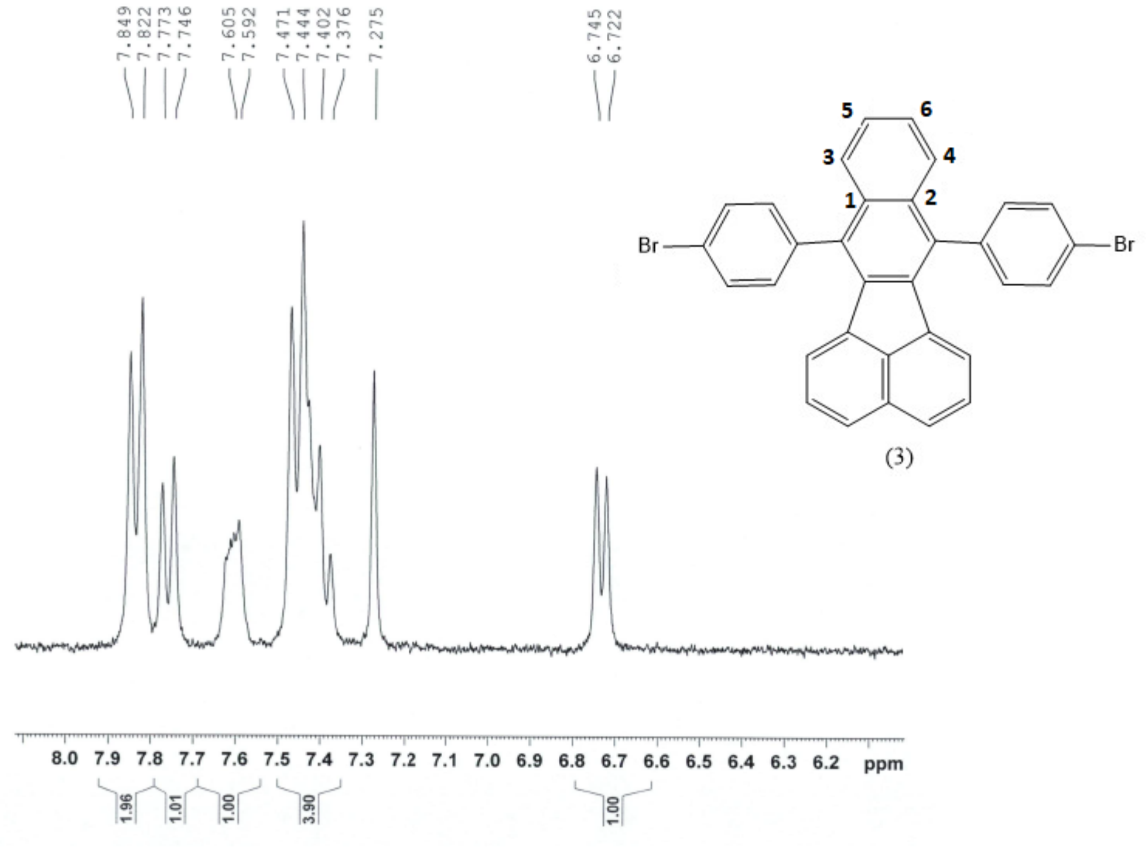

^{1}H NMR expand of **3**

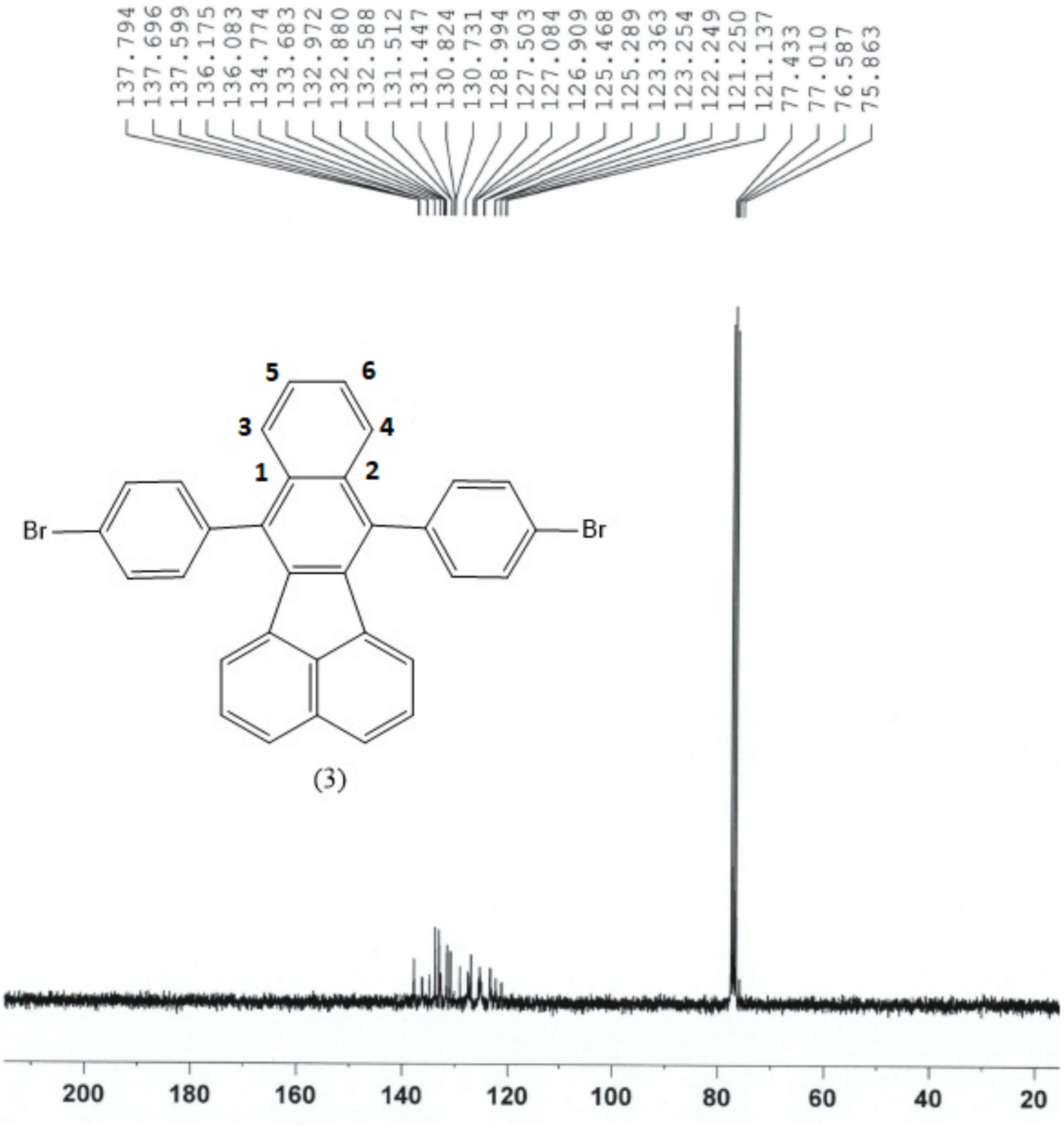

^{13}CNMR spectrum of **3**

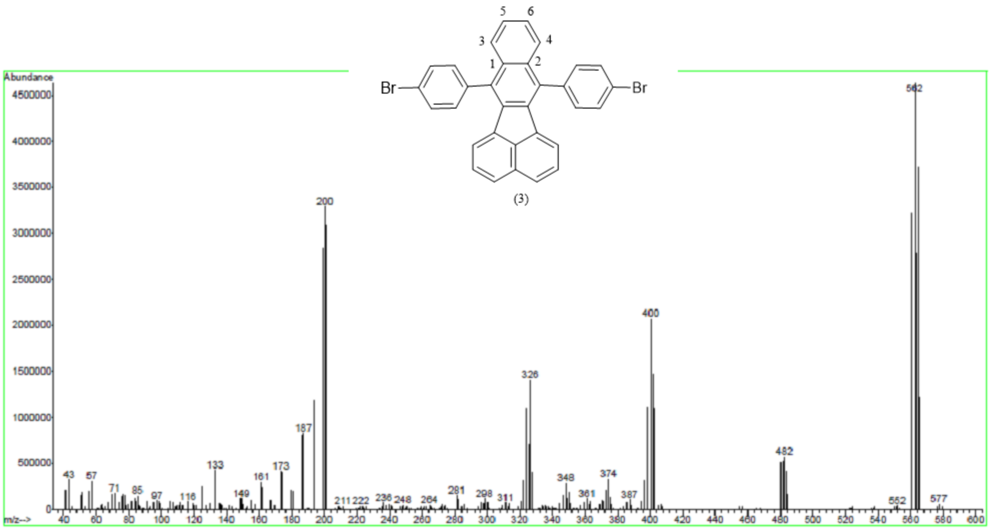

Mass spectrum of **3**

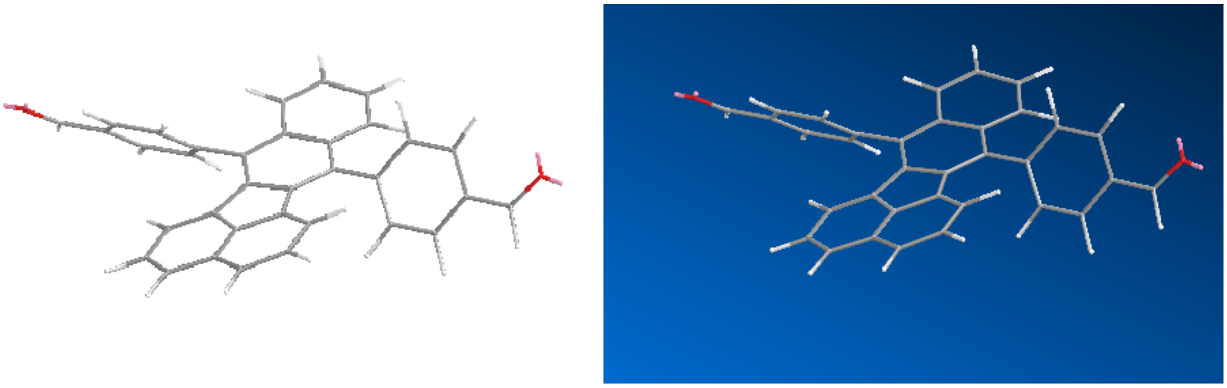

3D structure of **4**

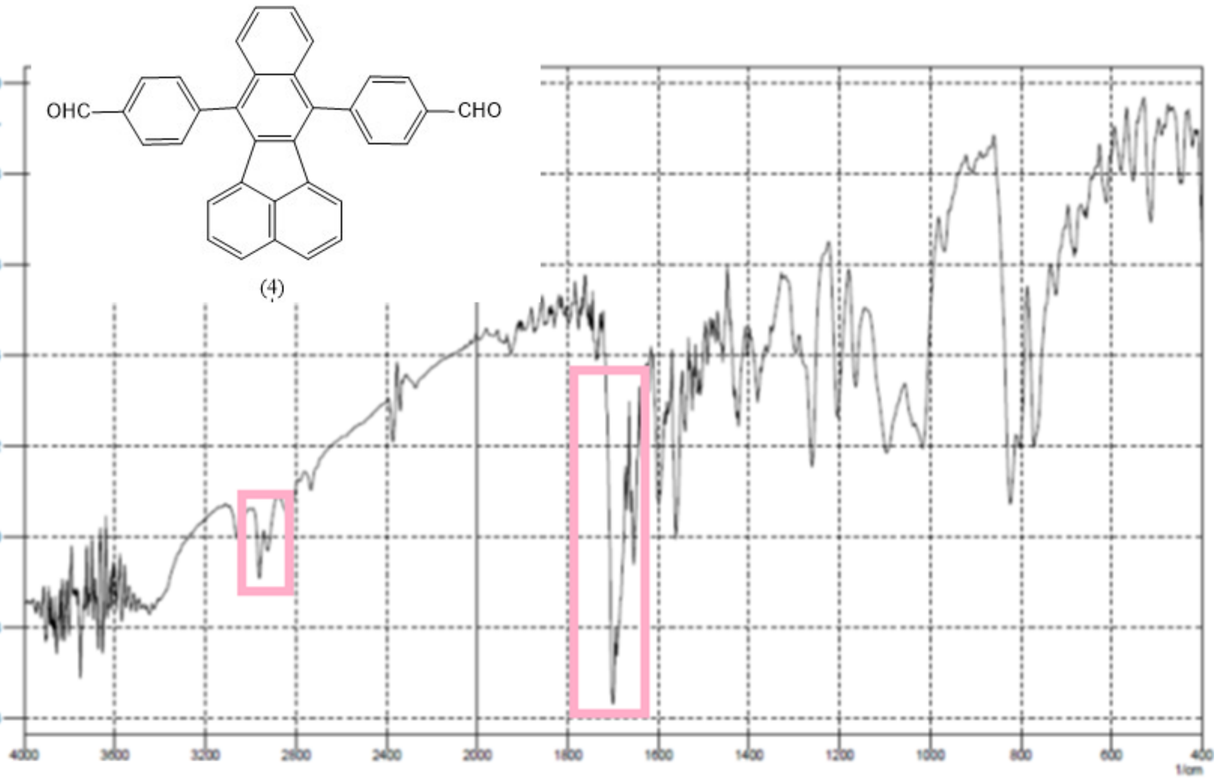

IR spectrum of 4,4'-(benzo[k]fluoranthene-7,12-diyl)dibenzaldehyde (**4**)

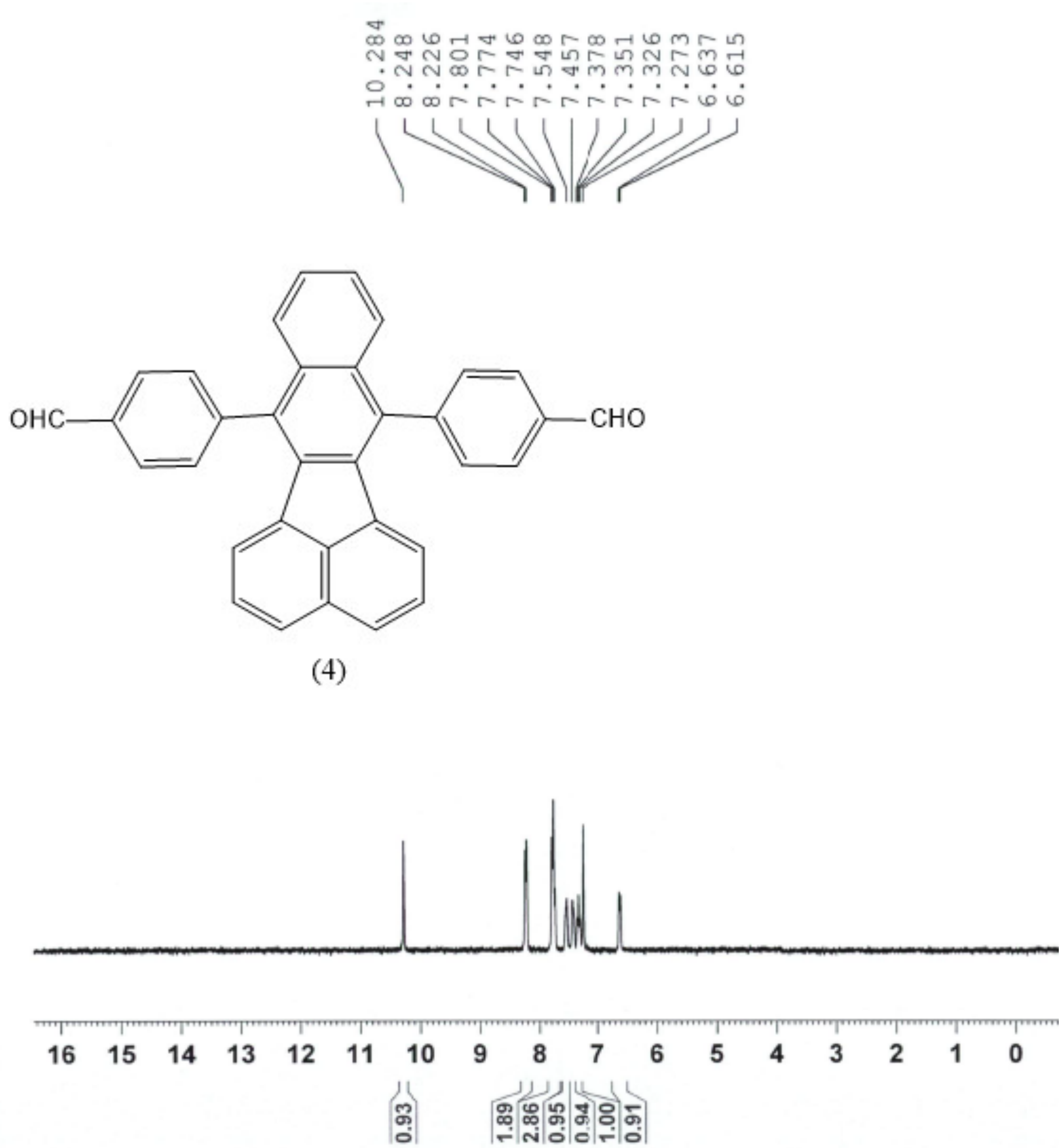

^{1}HNMR spectrum of **4** in $CDCl_3$

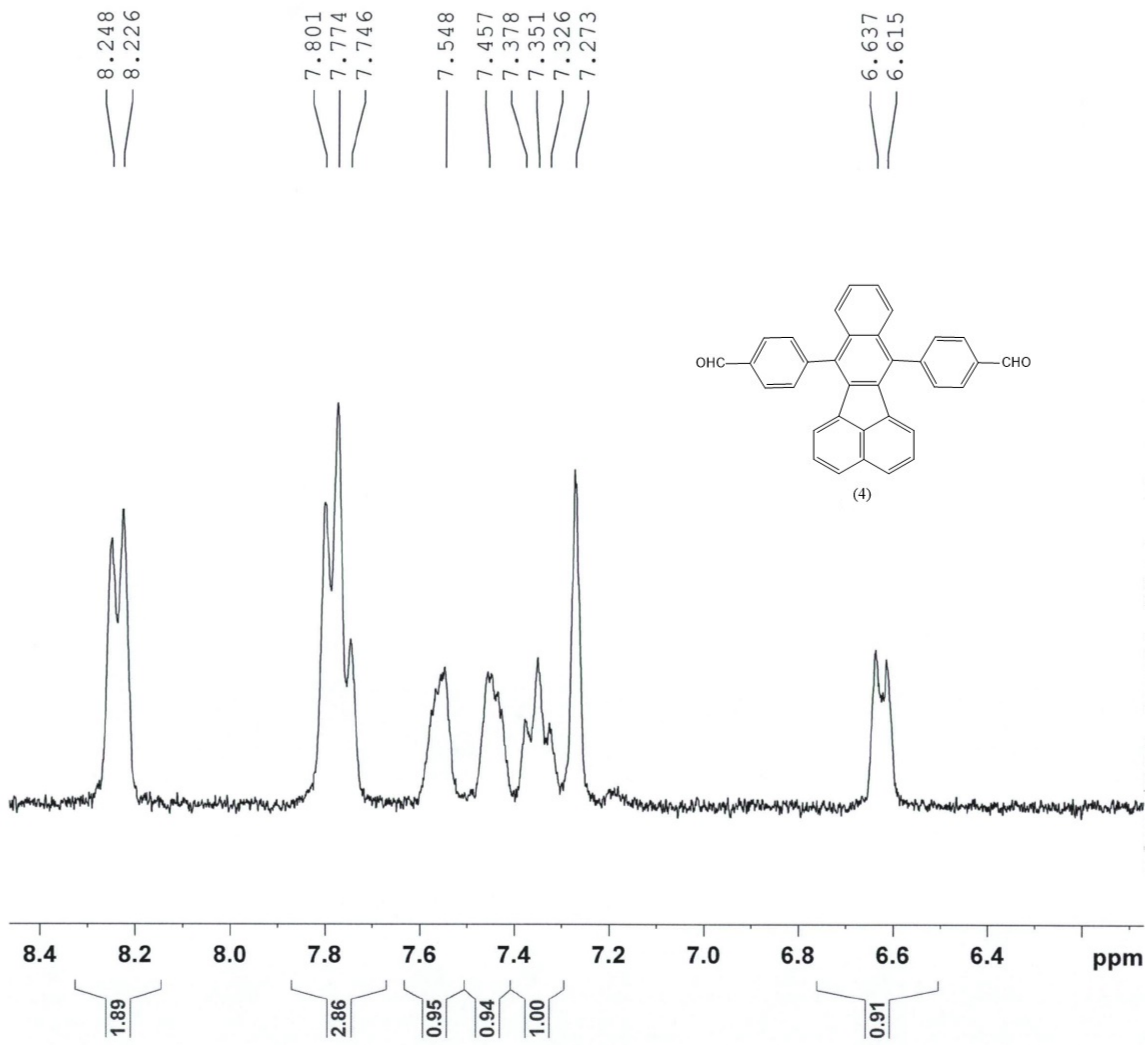

^{1}H NMR expand of **4**

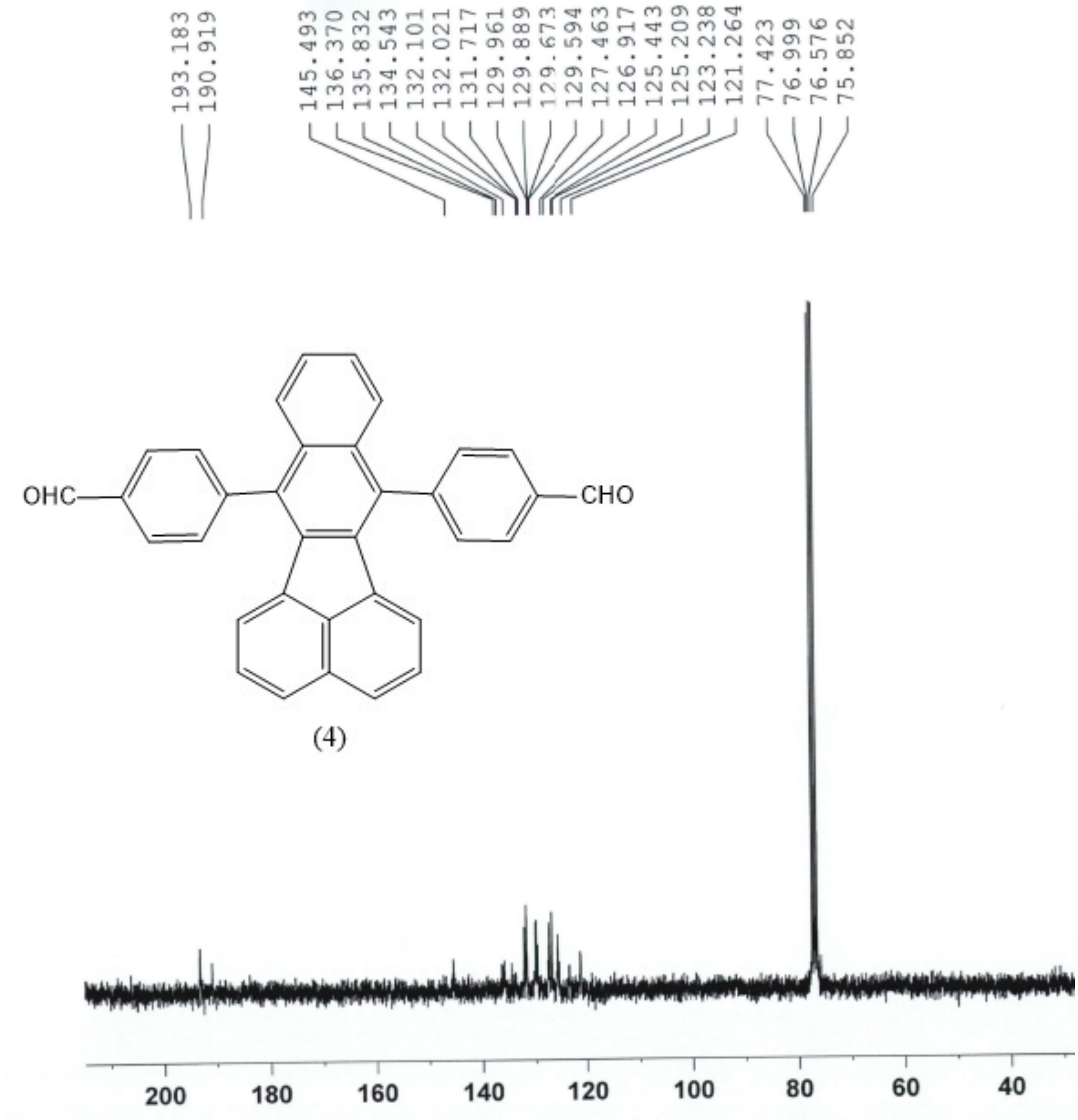

^{13}CNMR spectrum of **4**

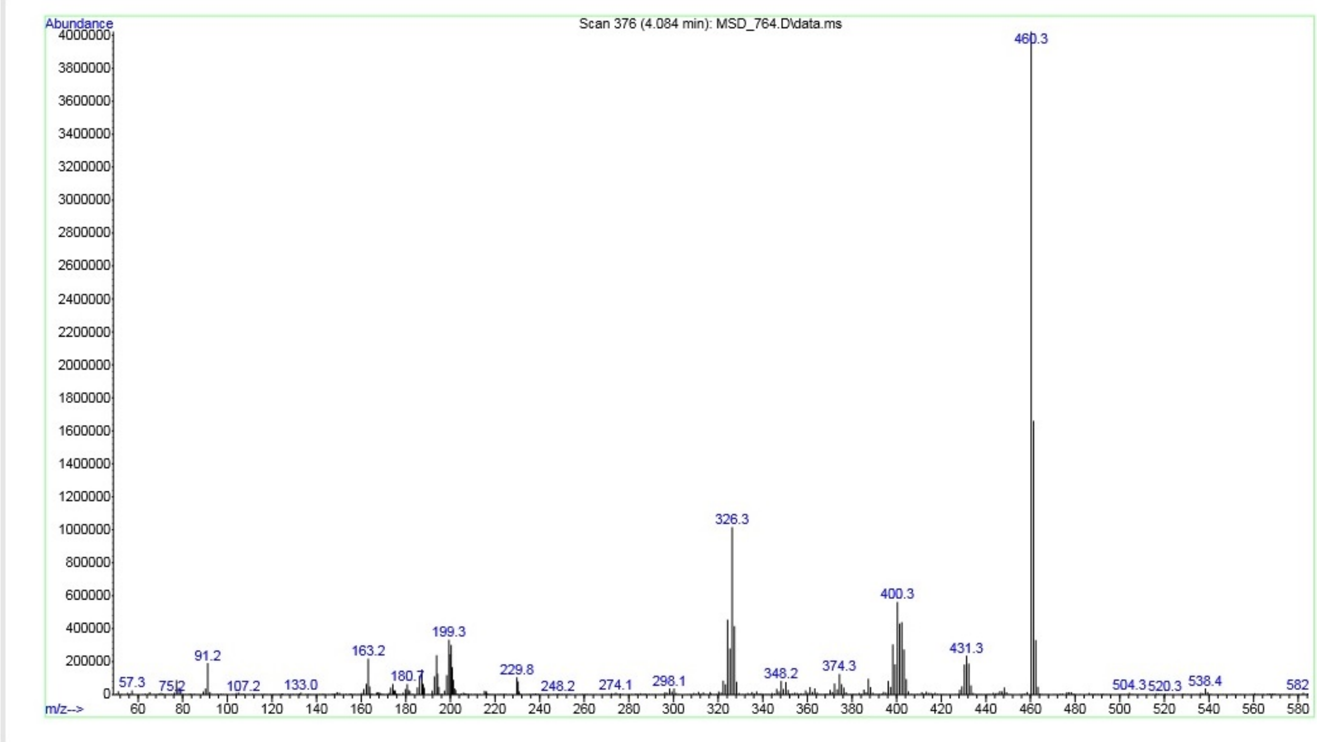

Mass spectrum of **4**

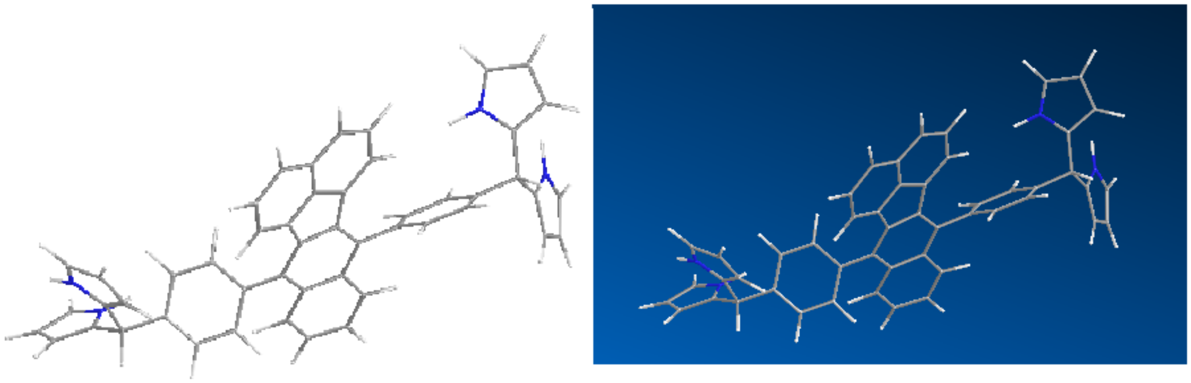

3D structure of **5**

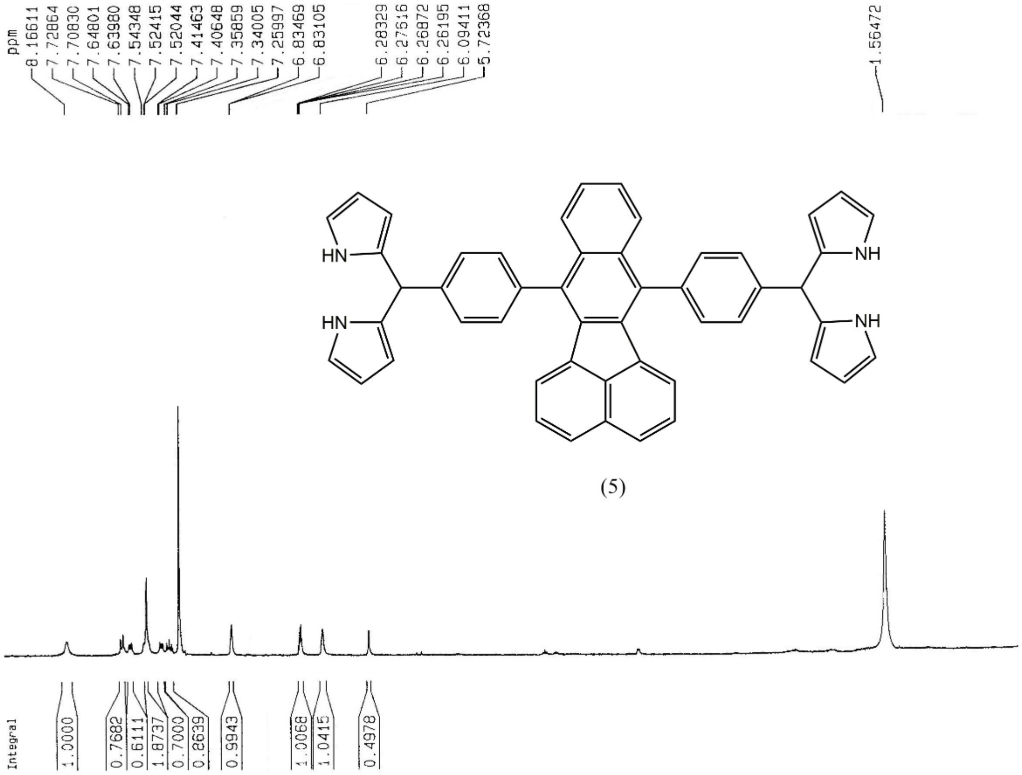

^{1}HNMR spectrum of **5** in $CDCl_3$

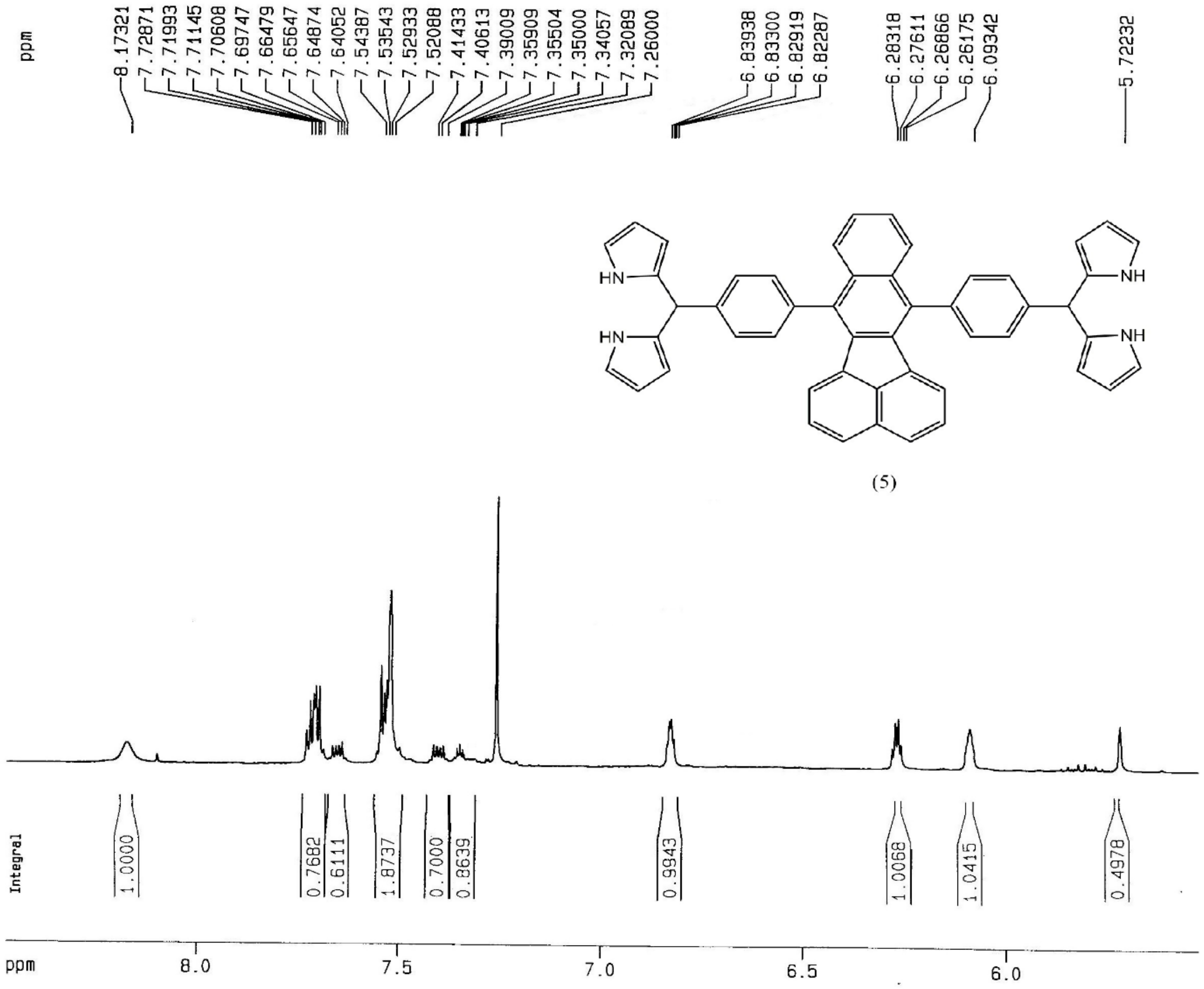

^{1}H NMR expand of **5**

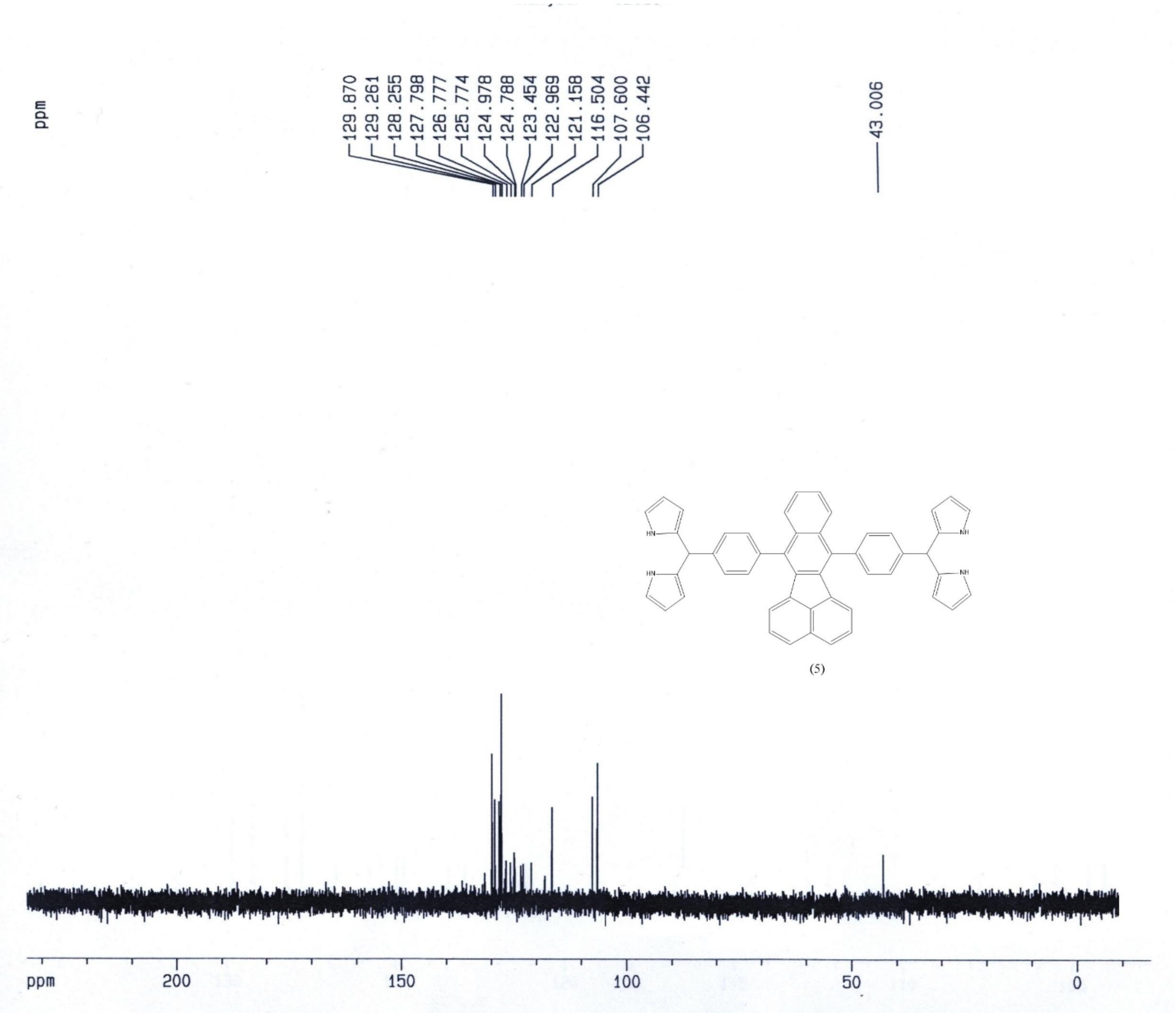

^{13}CNMR spectrum of **5**

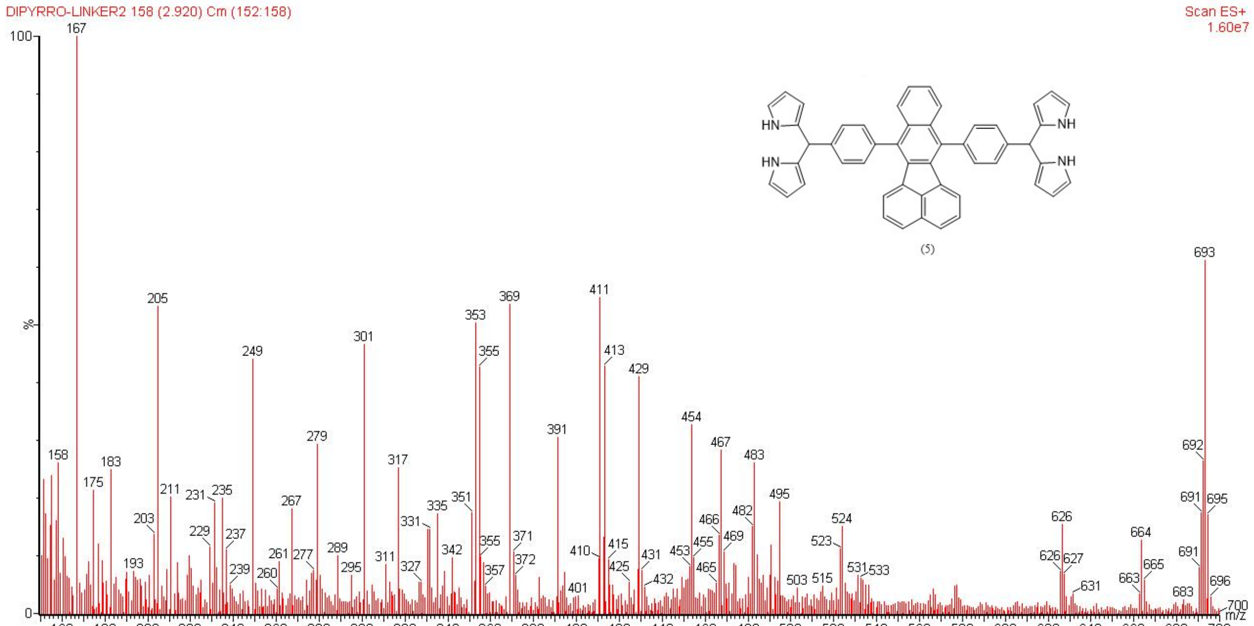

Mass spectrum of **5**

1. Bhandari, S.; Ray, S., A novel synthesis of bisbenzyl ketones by DCC induced condensation of phenylacetic acid. *Synthetic communications* **1998,** *28* (5), 765-771.
2. Keshtov, M.; Mal'tsev, E.; Pozin, S.; Marochkin, D.; Perevalov, V.; Blagodatskikh, I.; Petrovskii, P.; Khokhlov, A. In *Synthesis and photo-and electrophysical properties of conjugated copolyfluorenes with 7, 8, 10-triarylfluoranthene fragments in the main chain*, Doklady Chemistry, Springer: 2012; pp 23-29.
3. Farquhar, A. K.; Fitchett, C. M.; Dykstra, H. M.; Waterland, M. R.; Brooksby, P. A.; Downard, A. J., Diels–Alder Reaction of Anthranilic Acids: A Versatile Route to Dense Monolayers on Flat Edge and Basal Plane Graphitic Carbon Substrates. *ACS applied materials & interfaces* **2016,** *8* (35), 23389-23395.
4. Mata, J. A.; Falomir, E.; Llusar, R.; Peris, E., Preparation, properties and coordination of new conjugated ferrocenyl-based ligands with an end-capped nitrile. *Journal of Organometallic Chemistry* **2000,** *616* (1-2), 80-88.
5. Pye, C.; Fronczek, F. R.; Isovitsch, R., The Synthesis, Photophysical Characterization, and X-Ray Structure Analysis of Two Polymorphs of 4, 4′-Diacetylstilbene. *Helvetica Chimica Acta* **2010,** *93* (6), 1162-1171.
6. Paolesse, R.; Pandey, R. K.; Forsyth, T. P.; Jaquinod, L.; Gerzevske, K. R.; Nurco, D. J.; Senge, M. O.; Licoccia, S.; Boschi, T.; Smith, K. M., Stepwise Syntheses of Bisporphyrins, Bischlorins, and Biscorroles, and of Porphyrin− Chlorin and Porphyrin− Corrole Heterodimers. *Journal of the American Chemical Society* **1996,** *118* (16), 3869-3882.
7. Laha, J. K.; Dhanalekshmi, S.; Taniguchi, M.; Ambroise, A.; Lindsey, J. S., A scalable synthesis of meso-substituted dipyrromethanes. *Organic Process Research & Development* **2003,** *7* (6), 799-812.
8. Madhu, S.; Rao, M. R.; Shaikh, M. S.; Ravikanth, M., 3, 5-Diformylboron dipyrromethenes as fluorescent pH sensors. *Inorganic chemistry* **2011,** *50* (10), 4392-4400.
9. Zhu, S.; Bi, J.; Vegesna, G.; Zhang, J.; Luo, F.-T.; Valenzano, L.; Liu, H., Functionalization of BODIPY dyes at 2, 6-positions through formyl groups. *RSC Advances* **2013,** *3* (14), 4793-4800.
